# Antibiotic resistance in *Neisseria gonorrhoeae*: broad-spectrum drug target identification using subtractive genomics

**DOI:** 10.5808/gi.22066

**Published:** 2023-03-31

**Authors:** Umairah Natasya Mohd Omeershffudin, Suresh Kumar

**Affiliations:** 1 Post Graduate Centre, Management and Science University, University Drive, Off Persiaran Olahraga, Section 13, 40100 Selangor, Malaysia; 2 Faculty of Health and Life Sciences, Management and Science University, Seksyen 13, 40100, Shah Alam, Selangor, Malaysia

**Keywords:** antibacterial agents, drug development, drug resistance, iron-sulfur protein, Neisseria, proteome

## Abstract

*Neisseria gonorrhoeae* is a Gram-negative aerobic diplococcus bacterium that primarily causes sexually transmitted infections through direct human sexual contact. It is a major public health threat due to its impact on reproductive health, the widespread presence of antimicrobial resistance, and the lack of a vaccine. In this study, we used a bioinformatics approach and performed subtractive genomic methods to identify potential drug targets against the core proteome of *N. gonorrhoeae* (12 strains). In total, 12,300 protein sequences were retrieved, and paralogous proteins were removed using CD-HIT. The remaining sequences were analyzed for non-homology against the human proteome and gut microbiota, and screened for broad-spectrum analysis, druggability, and anti-target analysis. The proteins were also characterized for unique interactions between the host and pathogen through metabolic pathway analysis. Based on the subtractive genomic approach and subcellular localization, we identified one cytoplasmic protein, 2Fe-2S iron-sulfur cluster binding domain-containing protein (NGFG RS03485), as a potential drug target. This protein could be further exploited for drug development to create new medications and therapeutic agents for the treatment of *N. gonorrhoeae* infections.

## Introduction

*Neisseria gonorrhoeae* (gonococcus) is the etiological agent of gonorrhea, which causes the second most commonly occurring sexually transmitted infection. According to the Centers for Disease Control and Prevention, approximately 1.14 million infections are reported yearly [1]. However, it was also reported that about 550,000 estimated cases are due to treatment failure, particularly associated with the emergence of multi-drug-resistant gonorrhea strains [1]. Antibiotics' effectiveness in treating gonorrhea is dwindling due to the global spread of multi-drug-resistant strains.

It has been found that adolescents, the elderly, and men who have intercourse with other men are at high risk of acquiring gonorrhea. Although men who have intercourse with men are frequently identified as having this infection, the risk depends on specific sexual practices, making both genders vulnerable to this infection [2]. Urethritis is often a result of this infection in men and presents with purulent discharge from the urethra [3]. In comparison, infected women develop cervicitis and are frequently asymptomatic, although the infection can disperse to the urinary tract, leading to pelvic inflammatory disease [2,4]. Untreated infections can lead to severe epididymitis, salpingitis, pelvic inflammatory disease, ectopic pregnancy, and infertility.

This obligate human pathogen predominantly colonizes the mucosal epithelium of the reproductive tract. It causes infections via adherence to the mucosal epithelium, which is mediated by the bacterial pathogen's surface structures, which include type IV pili, opacity proteins, lipopolysaccharides, and porin [5]. Therefore, it is transmitted from an infected individual through direct human-to-human contact with the genital mucosa, anal mucosa, and oropharynx during sexual intercourse [6,7]. Untreated infections can lead to severe epididymitis, salpingitis, pelvic inflammatory disease, ectopic pregnancy, and infertility. Gonorrhea can also cause pregnancy complications and be passed on to children, resulting in blindness if left untreated.

This bacterial pathogen has shown a remarkable ability to develop resistance to nearly all antimicrobials used for treatment for approximately 70–80 years. Target alteration or reduction of target affinity is one of the critical resistance mechanisms in *N. gonorrhoeae*. In a recent study, the bacteria were found to develop resistance against extended-spectrum cephalosporin, cefixime, and ceftriaxone [8-12]. Cefixime is also no longer recommended as a first-line regimen [1].

Traditional drug development campaigns typically produce and test a few thousand compounds yearly, whereas computational technologies can accurately evaluate billions of molecules per week [12]. Thus, ever-growing efforts in the current biomedical arena utilize computational-aided drug design or *in silico* analysis to accelerate drug design and development [8]. Many studies have successfully identified potential drug targets and vaccine candidates using *in silico* methods [9-11,13-17].

Recent research employing a subtractive genomics strategy for many pathogenic strains has reported the successful identification and discovery of novel species-specific treatment targets. Subtractive genomics is defined as the method of removing host homologous proteins from the proteomes of the host and pathogen [18,19]. The technique is utilized to analyze the entire proteomes of the host and pathogen to identify proteins with unique therapeutic properties that are present only in the genome of the pathogen. In this study, a subtractive genomic approach was applied to identify potential drug candidates in the core proteome of *N. gonorrhoeae* (12 strains).

## Methods

### Retrieval of the core proteome from the EDGAR 3.0 database

The core proteome of *N. gonorrhoeae* (12 strains) was retrieved via EDGAR 3.0 database [20]. The list of 12 *N. gonorrhoeae* strains analyzed in this study is MS11 NC 022240, NCCP11945 NC 011035, FDAARGOS 205 NZ CP020418, FA 1090 NC 002946, 32867 NZ CP016015, 34530 NZ CP016016, 34769 NZ CP016017, 35 02 NZ CP012028, FA19 NZ CP012026, FA6140 NZ CP012027, FDAARGOS 204 NZ CP020415, and FDAARGOS207NZCP020419. The reference strain, *Neisseria_gonorrhoeae*_MS11_NC_022240, and the core proteome that showed hits against the reference strain were further investigated to predict new potential drug targets for *N. gonorrhoeae*.

### Identification of non-paralogous protein sequences

CD-HIT tools filter the paralogous sequences of the bacterial proteins [21]. The algorithm parameters are set to a sequence identity cutoff of 0.6 (60%), a bandwidth alignment of 20 amino acids, and the exclusion of sequences <100 amino acids in length, as proteins that have <100 amino acids could not be interpreted as essential to the pathogen’s survival. The threshold to remove genes with 60% similarity is considered to eliminate paralogues; therefore, any protein identified above the value was excluded.

### Identification of proteins containing essential genes

The Database of Essential Genes (DEG) consists of an extensive list of all organisms' essential genes, including *N. gonorrhoeae* [22]. To identify essential genes that are indispensable for the survival of the bacteria, the identified proteins were subjected to BLASTp against the deposit data of DEG. The E-value was set to <0.0001 [13,23].

### Identification of proteins containing virulence factors

Virulence factors (VFs) serve as a crucial determinant influencing pathogenicity. The Virulence Factor Database (VFDB) contains VFs from 25 important bacterial pathogens and is used to identify virulent proteins [24]. The proteins were subjected to BLASTp against the core dataset of VFDB. The E-value was set to <0.0001 with an alignment cutoff value of 1%.

### Identification of protein sequences non-homologous to the human proteome

To identify non-homologous proteins of pathogens relative to the human host, a BLASTp search was applied to align the identified virulent proteins that were non-homologous to the human proteome. This step is crucial to prevent unintentional binding with proteins crucial to the host. The proteins identified were subjected to BLASTp against the human proteome (*Homo sapiens*; 9606; UP000005640) downloaded from UniProt [25]. The E-value was set to >0.005, and the sequence identity to <50% [26]. Non-homologous proteins were defined as those that demonstrated "hits" at or above the thresholds, as has been described elsewhere [27-29].

### Identification of protein sequences non-homologous to human gut microbiota

The protein sequences were then subjected to BLASTp against human (*Homo sapiens*; 9606) gut microbiota proteins with an E-value of > 0.005 to identify proteins that shared a high degree of similarity with the human gut bacteria. Proteins that shared high similarity were excluded. The cutoff values were set to default based on the parameters described above in section “Identification of protein sequences non-homologous to the human proteome.”

### Identification of anti-target proteins

Drugs are designed to bind to and inhibit the proteins of pathogens. However, these compounds might unintentionally bind to proteins crucial to the host proteins' bio-cellular processes, leading to unintended pharmacokinetic effects. Such proteins are termed anti-targets. Anti-targets in humans include the ether-a-go-go-related gene (hERG), the pregnane X receptor, the constitutive androstane receptor, and P-glycoprotein [30]. The identified proteins were subjected to BLASTp against human anti-target proteins from the NCBI database with an E-value > 0.005, and a similarity threshold of <50% was used to screen anti-target proteins. Proteins that showed similarity values <50% were included [8].

### Broad-spectrum analysis

A broad-spectrum analysis identifies homologous proteins in multiple bacterial pathogens [9]. To identify broad-spectrum proteins, non-homologous proteins were aligned with BLASTp against a wide range of pathogenic organisms retrieved from the EMBL-European Bioinformatics Institute (EBI) database [31]. The E-value was set to <0.0001.

### Host-pathogen interactions

The non-homologous proteins were computed with BLASTp against databases Host-Pathogen Interaction Database (HPIDB version 3.0), Pathogen Host Interactions (PHI-base version 4.2), Pathogen-Host Interaction Search Tool (PHISTO version 2) of proteins that exhibit host-pathogen interactions. Host-pathogen interaction analysis is essential for identifying pathogenic proteins that show interactions with the human host. The E-value was set to <0.0001 and an alignment cutoff of 1% was used [32-34].

### Analysis of unique metabolic pathways of *N. gonorrhoeae*

Metabolic pathway analyses of *N. gonorrhoeae* were performed using the Kyoto Encyclopedia of Genes and Genomes (KEGG) database [35]. The listed host (*Homo sapiens*) metabolic pathways were compared against the pathogen to identify unique pathways present only in the pathogen. The query proteins were then functionally annotated by BLASTp in the KEGG Automation Annotation Server (KAAS) against the KEGG database. The KEGG orthologs (KOs) of the identified metabolic proteins were assigned by the bi-directional best hit method in KAAS [36]. Proteins involved in unique metabolic pathways of the pathogen were further analyzed.

### Sub-cellular localization

The subcellular localization was identified using PsortB 3.0 [37]. Proteins localized in the cytoplasm are viable drug targets, while membrane proteins are often targeted as vaccine candidates [12]. Membrane proteins are likely to secrete antigenic proteins that the immune system can detect, which is why they are preferred as potential vaccine candidates [38].

### Druggability analysis of the identified non-homologous protein sequence of *N. gonorrhoeae*

A protein must be druggable to be classified as a potential drug target. DrugBank provides comprehensive drug information comprising molecular information on thousands of Food and Drug Administration–approved drugs, nutraceutical drugs, and experimental drugs [39]. To perform the analysis, the proteins were screened by performing BLASTp with an E-value < 0.0001. Proteins that showed significance against the core dataset of the Drug Bank database were identified as druggable targets.

To further screen ideal drug target candidates for *N. gonorrhoeae*, the proteins were further filtered based on the 10 rules of druggable proteins that are desirable to human targets [40,41]. The 10 drug target properties are: molecular weight < 100 kDa, hydrophobicity between –0.150 and –0.350, length between 400 and 600 amino acids, the signal motif is present, no PEST motif, more than 2 N-glycosylated amino acids, not more than one O-glycosylated serine, a mean pI of <7.2, presence of a transmembrane helix, and a cytoplasmic membrane location [40]. The physicochemical parameters were predicted using ProtParam tools in the ExPassy server to calculate amino acid length, hydrophobicity, and theoretical pI [42]. To annotate signal peptides, the SignalIP program was used (http://www.cbs.dtu.dk/services/SignalP/) [43]. The presence of a transmembrane helix (THMM) was analyzed by performing the TMHMM method ((http://www.cbs.dtu.dk/∼Krogh/TMHMM/) [44]. PEST regions were identified as a sequence of amino acids containing more than 12 P, E, S, or T residues. These regions were identified by using (http://emboss.cbr.nrc.ca/cgi-bin/emboss/epestfind). The NetOglyc program was used to analyze O-glycosylation (https://services.healthtech.dtu.dk/service.php?NetOGlyc-4.0), and a similar program was used to identify N-glycosylation (https://services.healthtech.dtu.dk/service.php?NetNGlyc-1.0) [45]. Proteins with relatively high target-like properties were selected for druggability analysis.

### Functional annotation of protein sequences

Functional annotation was performed using UniProt, Gene Ontology (GO), Pfam, and PROSITE. The UniProt knowledgebase contains many protein sequences and comprehensive annotations [25,46-48]. The GO project (http://www.geneontology.org/) generates structured, regulated vocabularies and categories for annotating genes, gene products, and sequences [46,49]. Pfam (http://pfam.xfam.org/) provides information on protein families. The domain database is frequently used to analyze novel genomes and metagenomes and drive experimental work on specific proteins and systems, with a collection of 12,000 families that experimental and computational biologists use extensively throughout the biological sciences [50]. PROSITE (http://prosite.expasy.org/) is a collection of documentation entries that describe protein domains, families, and functional sites and the patterns and profiles used to identify them [51]. Two signatures are used to identify these regions: generalized profiles (weight matrices) and modular protein domains (regular expressions). Regular expressions denote short sequence motifs that frequently correspond to functional or structurally significant residues [48].

### Homology modeling and protein evaluation

Identified proteins were homology-modeled to obtain the 3D structures using the SWISS-MODEL server (swissmodel.expasy.org) [52]. The homology model was built through a hidden Markov model based on the aligned target and template in the SWISS-MODEL Template Library. The proteins that were 3D-modeled using SWISS-MODEL were evaluated using PyMOL [53].

### Validation of protein structure

The PROCHECK suite of tools checks the stereochemistry of a protein structure in detail. It produces several charts in PostScript format and a detailed residue-by-residue list. These measure the structure's quality compared to similarly refined structures of the exact resolution [54]. The modeled 3D-structured proteins, with stereochemical and structural information, were evaluated using PROCHECK. Protein Structure Analysis (ProSA) is a popular tool for checking 3D models of protein structures for mistakes. The homology-modeled protein sequence structure was accessed using the ProSA server based on the calculated Z-score [55].

## Results and Discussion

**Fig. 1.**
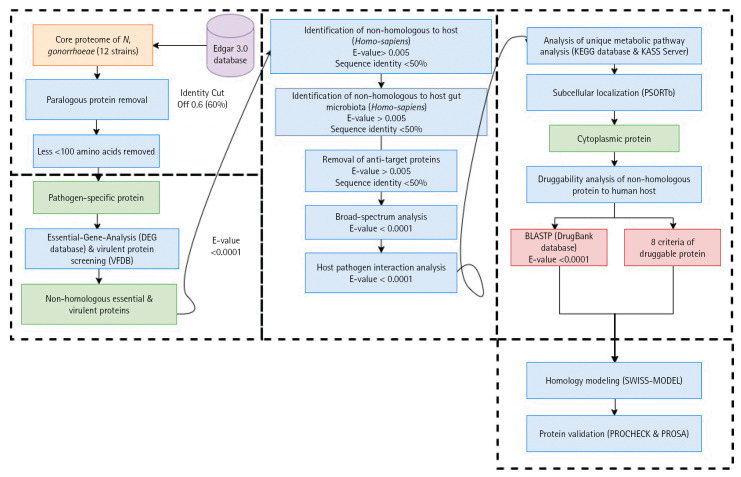
Schematic workflow of the identification of potential drug targets among the core proteome of all 12 *Neisseria gonorrhoeae* strains. KEGG, Kyoto Encyclopedia of Genes and Genomes; KASS, KEGG Automatic Annotation Server.

In this study, we explored potential drug target candidates for the core proteome of *N. gonorrhoeae*. We employed a subtractive genomics approach to screen potential drug targets [7,9,56]. The schematic workflow and analysis summarization can be referred to in Table 1 and Fig. 1.

### Subtractive genomic analysis

The core proteome refers to proteins shared in all strains that are consistently used in various circumstances [57]. Therefore, the core proteome would be beneficial to reveal broad-spectrum candidates of this pathogen. To identify shared and unique features of the protein, we downloaded a total of 84,460 protein sequences inclusive of all *Neisseria* species from the EDGAR 3.0 database, from which we selected the core proteome sequences of 12 strains of *N. gonorrhoeae*, containing 12,300 sequences.

All core proteome sequences of *Neisseria* species were submitted to CD-HIT to remove paralogous sequences. The removal of paralogous sequences is one of the first steps in the subtractive genomics approach. Paralogous genes are found in a single organism correlated by a gene duplication event [58]. When compared to other organisms, identifying loci and real single-nucleotide polymorphisms from short sequences, especially bacteria, remains difficult for species with duplicated genomes, as duplicated sequences might be incorrectly grouped into a single locus, making valid allelic variation identification difficult [59]. Assembly approaches that use sequence similarity thresholds to identify homology may overlap the paralogous genes.

The overlapping of paralogues increases sequence variation at those loci, which may or may not affect species relationships [60-62]. Still, it is predicted to result in underestimated branch lengths. Moreover, various studies have proved that proteins possessing a sequence identity greater than 60% are paralogous to each other. Thus, paralogues that shared >60% identity were excluded from this analysis. Based on the report, 944 proteins were identified as non-paralogous. Furthermore, proteins containing <100 amino acids were excluded, as these proteins are unlikely to carry essential genes for the survival of bacterial pathogens [14]. Out of the 944 initial proteins, only 476 proteins of *N. gonorrhoeae* species were included for further analysis.

Essential genes are indispensable for carrying out a bacterial pathogen's cellular processes. The essential genes are preferably developed as potential drugs, as antibacterial compounds are generally designed to target and inhibit these essential genes [8]. Targeting these proteins could disrupt the bacteria's protein functionality, which would be beneficial for drug discovery. Based on the analysis, 421 proteins showed significant hits against a deposited dataset of bacteria containing essential genes in DEG.

Exploring VFs and identifying novel VFs of *N. gonorrhoeae* is a significant contribution, as VFs play a vital role in the modulation or degradation of the host defense mechanism [63]. The VFDB server reported 120 proteins as virulent proteins. The identified VFs can be further explored as important targets to inhibit the pathogenicity of *N. gonorrhoeae*.

Based on the screening of non-homologous proteins, 101 proteins showed no hits against the human proteome. The exclusion of homologous proteins from the human proteome is crucial in subtractive genomics analysis, as these can result in adverse pharmacokinetics through cross-reactivity [64].

Antibiotic interactions depend on the gut microbiota's ecological system [65]. Proteins that share similarities with the human gut microbiota will interrupt the typical flora environments of the gut during drug interactions. The analysis of non-homologous proteins against human gut microbiota resulted in 95 proteins having a percentage identity of <50%. These proteins were identified as non-homologous and were further explored, as they are unlikely to contribute to cross-reactivity during drug interactions.

Numerous drug candidates have been pulled from the market due to carcinogenicity; thus, cross-reactivity and carcinogenicity testing is critical for building an effective pharmacological molecule [10]. Although non-homologous host proteins for this pathogen were deleted from the non-paralogous sequences, anti-target analysis was conducted to avoid harmful effects caused by inadvertent binding of medications administered to treat the pathogen to host anti-targets. The anti-target analysis identified 41 targeted proteins.

An ideal potential drug candidate can be utilized for multiple infections in a future setting. A protein is considered as a potential target for broad-spectrum drugs if a non-homologous protein is present in more than 25 bacterial pathogens [66]. All 41 proteins screened were identified as broad-spectrum through the broad-spectrum analysis.

### Host-pathogen interactions and metabolic pathway analysis

A metabolic pathway is a series of processes or relationships among genes and their metabolites that result in the synthesis or modification of a system component required for the proper functioning of a biological system [67]. Based on the targeted query proteins, 27 proteins were identified to have unique host-pathogen interactions. Therefore, these proteins were analyzed to elucidate the metabolic pathways involved. The KAAS server of KEGG provides molecular network information on targeted molecules [68]. The server is used to characterize and identify unique metabolic pathways through a comparison between *N. gonorrhoeae* and human pathways [69]. The comparative analysis of the metabolic pathways between humans and *N. gonorrhoeae* identified 14 distinct pathogen-specific metabolic pathways. Based on this analysis, three of the 27 proteins were identified as KEGG orthologous and involved in three unique pathways of *N. gonorrhoeae*, which can be employed as new treatment targets (Table 2). The analysis revealed 27 human metabolic pathways, 36 *N. gonorrhoeae* pathways, and 14 unique-pathogen-specific pathways (Supplementary Table 1).

The pathways were assigned KO identifiers, where each KO assignment provides molecular functions (MFs) in the KO (KEGG Orthology). The three essential proteins were found to be engaged in the following metabolic pathways: O-antigen nucleotide sugar biosynthesis (KO: K00523), lipopolysaccharide (LPS) biosynthesis (KO: K02535), and nicotinate and nicotinamide metabolism (KO: K08324). The results of this analysis included both biosynthesis and metabolism pathways.

Metabolism pathways involve metabolic interactions in which a molecule is changed to another chemical through a sequence of processes aided by specific enzymes. Biosynthetic metabolism, sometimes referred to as anabolism, is the process by which macromolecules are synthesized from specified building blocks and these processes are mostly multi-enzymatic in nature [70].

LPS is one of the key ingredients of Gram-negative bacteria's outer cell walls, and it plays a crucial role in the pathogen's survival. The enzyme UDP-3O-[3-hydroxymyristoyl] N-acetylglucosamine deacetylase [EC:3.5.1.108] (LPxC) catalyzes the second step in the production of lipid A, which forms LPS structures [71]. It is a unique amphiphilic molecule found in the outer membranes of practically all Gram-negative bacteria. LpxC inhibitors may be used as antibiotics.

O-antigen is located on the outer membrane of Gram-negative bacteria and is composed of repeat-unit polysaccharides. It is the immunodominant component of LPS and is the easiest target for the host's humoral response [72]. It functions as a bacteriophage receptor and is a dependable indicator of potential virulence [73]. O-antigen modification can overcome the host's defense mechanisms and influence the stages of the infection [72]. Nucleotide sugar biosynthesis is the first of three gene cluster groups on the O-antigen chromosome [73]. CDP-4-dehydro-6-deoxyglucose reductase, E3 [EC:1.17.1.1], is a critical biosynthetic precursor for structural variation in O antigen [74]. Furthermore, O-antigen was predicted to be a virulent protein (NGFG RS03485).

Nicotinate (niacin) and nicotinamide are coenzymes that are precursors to nicotinamide adenine dinucleotide (NAD^+^) and nicotinamide-adenine dinucleotide phosphate (NADP^+^) [75]. NAD is a cofactor that is required by all living organisms. Each bacterial species has its mechanism for reducing NAD^+^ to NADH, such as respiration, glycolysis, the tricarboxylic acid cycle, or fermentation [76]. The enzyme succinate semialdehyde dehydrogenase [EC:1.2.1.16] catalyzes the reduction of NAD^+^ to NADH and produces a succinate substrate for the tricarboxylic acid cycle. It is similar to succinate-semialdehyde dehydrogenase [EC 1.2.1.24].

Inhibiting these enzymes identified in the metabolic pathways may disrupt critical processes for *N. gonorrhoeae* survival and virulence, and thus may be a viable antibacterial therapy method. Given that each antibiotic has a limited duration of action, and resistance will eventually develop, mainly if the same enzymes are repeatedly targeted, it is critical to create new classes of inhibitors that target previously untargeted cellular enzymes to maintain control of infectious diseases.

### Identification of druggable proteins of *N. gonorrhoeae*

Druggable proteins are characterized as binding with small molecules, thereby inhibiting protein functions [18]. Criteria for distinguishing between a suitable drug candidate and a vaccine candidate are based on the protein's subcellular localization, druggability, and physicochemical properties. Subcellular localization is a critical aspect of therapeutic targets that helps understand the protein functionality, as many proteins exist in several locations. The localization of the three proteins involved in the pathogen-specific pathways was analyzed and achieved using PsortB v.3.0.

Based on the analysis performed, all three proteins were present in the cytoplasm, which signifies that the proteins are potential drug target candidates. Cytoplasmic proteins are often considered potential drug targets for developing small-molecule drugs. The analysis using the DrugBank database showed that these cytoplasmic proteins were all druggable. The drug name, drug bank ID, and E-value score are detailed in Supplementary Table 2.

The physicochemical properties of a drug play a significant role in drug development and serve as a critical criterion of drug candidates. Most therapeutic projects consistently use the following criteria: molecular weight < 100 kDa, hydrophobicity between –0.150 and –0.350, length between 400 and 600 amino acids, the signal motif is present, more than one N-glycosylated amino acids, not more than one O-glycosylated serine, a mean pI of <7.2, the presence of a transmembrane helix, and a cytoplasmic membrane location. Although these criteria are generally used for potential drug targets, they are not absolute requirements.

Ideal drug target candidates should have low molecular weight, increasing the drug molecules' absorption rate. The isoelectric point (i.e., the mean pI) indicates whether amino acids are acidic or basic. Proteins should be acidic based on the drug target criteria, as antibiotics interact differently based on the acidity level. Based on the analysis using standard criteria for suitable drug targets, the proteins identified included NGFG RS03485. The proteins were found to have relatively high drug target properties, as shown by a molecular weight < 100 kDa, mean pI < 7.2, hydrophobicity between –0.150 and –0.350, length between 400 and 600 amino acids, at least one O-glycosylation, and more than two N-glycosylated amino acids (Table 3).

### Functional annotation characterization of identified proteins

To better understand protein functionality, we performed functional annotations and GO characterization by identifying proteins against the core dataset of the GO and Uniprot databases. The functional annotation of proteins provides a molecular understanding that will benefit the drug development process and provide potential antibacterial targets. The analysis showed that the protein NGFG RS0385 harbors the cluster 2Fe-2S iron-sulfur cluster binding domain-containing protein (Supplementary Table 3).

The GO analysis identified three primary MFs of NGFG RS03845: electron transfer activity, metal ion binding, and iron-two-sulfur cluster binding (2Fe-2S). The MFs indicate behaviors rather than the entities (molecules or complexes) that conduct the actions and do not describe where, when, or in what context the activity occurs [49].

Proteins that engage in the 2Fe-2S function, also known as ferredoxins, are iron-sulfur proteins that enable the biological generation or usage of hydrogen gas by bacteria by acting as an electron-mediating catalyst [77]. This iron protein was initially isolated from the saccharolytic anaerobe *Clostridium pasteurianum*. Its structure was characterized by automated Edman degradation of the entire protein and peptides derived from tryptic and staphylococcal protease digestion [78]. An essential ancestor of all iron-sulfur proteins, ferredoxins are found mostly in anaerobic bacteria such as *Escherichia coli* and have a low molecular weight (6,000 Da) and two (4Fe-4S) clusters, which indicate that they evolved in the absence of oxygen [79]. Iron-sulfur cluster proteins play a significant role in bacterial pathogenesis, acting as an inherent VF [80].

Numerous transcriptional regulators in bacteria, including many mammalian pathogens, require iron-sulfur clusters as essential cofactors. Clusters are sensitive to iron availability, oxygen tension, and oxygen and reactive nitrogen species. They allow bacteria to swiftly change their gene expression profiles in response to changing environmental conditions [80]. The 2Fe-2s cluster comprises two iron atoms and two inorganic sulfur atoms as bridge ligands [81].

Several studies have shown that redox metabolism is viable when designing anti-infectious medications, and iron-sulfur proteins have been specifically implicated as a promising target [82]. The role of Fe-S cluster repair in the survival of *Yersinia pseudotuberculosis* in the spleen, as determined by a previous study, suggests that extracellular bacteria may rely on this pathway for survival within host tissues [83]. Another study found that iron-sulfur clusters were identified from *N. meningitidis*, which had structural homologies with *Vibrio cholera* toxin and enterotoxin from *E. coli*. The observation led to the inhibition of the iron-sulfur cluster, causing a loss of ASP-ribosyltransferase enzymatic activity [84].

Moreover, to colonize host tissues successfully, bacteria must respond to and detoxify numerous host-derived antimicrobial chemicals, such as nitric oxide (NO). NO has direct antibacterial activity by targeting proteins containing iron-sulfur clusters [83]. This finding suggests that the iron-sulfur cluster binding domain-containing identified in this study can be further explored as a promising drug target for *N. gonorrhoeae*.

An electron transport chain (ETC) is an electron carrier sequence that moves electrons from donors to terminal electron acceptors. As *N. gonorrhoeae* is an obligate human pathogen, the pathogen can receive electrons from oxygen and nitrate, yet its ETC has not been extensively explored. One study comprehensively determined these redox proteins' functions [84]. Ideally, more studies should explore the ETC as the antibacterial target for *N. gonorrhoeae*.

Metal-binding proteins chelate metal ions [85]. The chelation of metal ions commonly involves histidine or cysteine. In some circumstances, this is required for folding and tertiary structure maintenance, which signifies the survival of the bacterial pathogen. One of the genes identified as metal binding-specific is the mntABC transporter identified in *N. gonorrhoeae*. This finding reveals that the growth of *N. gonorrhoeae* could be driven by either manganese or zinc ions, indicating that the mntABC system could transport both ions and significantly affect pathogen survival [86]. Inhibiting the metal-binding proteins could disrupt the growth of *N. gonorrhoeae*.

An exotoxin is a bacterial toxin that can harm the host by killing cells or interrupting normal metabolism. A bacterial exotoxin, also known as a bacterial ADP-ribosylating exotoxin, acts by depositing the ADP-ribose moiety of NAD onto eukaryotic target proteins [87]. Protein toxins are the predominant virulence agents of many bacterial species, making them potential therapeutic targets. A major problem in the 21st century is the emergence of resistant strains of bacteria. However, these treatments put less selection divergence on bacteria and are less likely to cause resistance [88]. Secondly, even after the bacteria have been eliminated from the host, symptoms may persist if the toxin remains there [89]. Thirdly, non-antibiotic treatments avoid the disruption of normal microbiota sometimes associated with antibiotic treatments [90].

In contrast, methionine N-acyltransferase acts as a catalyst in the acetylation of L-methionine to N-acetyl-L-methionine. The N-acetyl-L-methionine present in bacteria acts as a translation initiator [91]. The consistent findings in our study suggest that this may be a novel antibacterial target.

### Homology modeling and validation of 3D protein structure

**Fig. 2.**
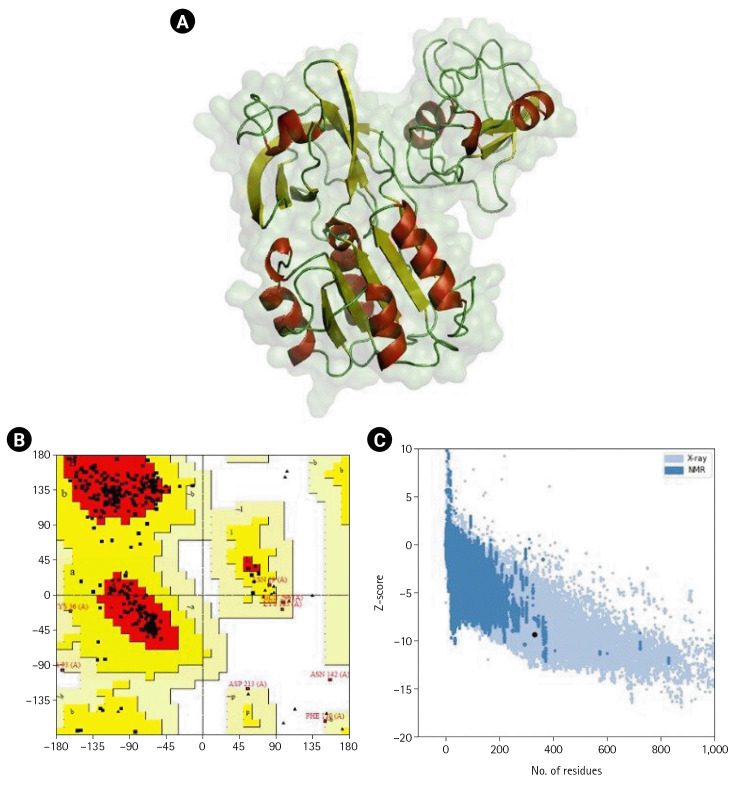
(A) 3D-modeled structure of NGFG RS03485 evaluated using PyMOL. Red: α-helices, yellow: β-sheets, and green: loops. (B) Ramachandran plot analysis of the 3D-modeled structure of NGFG RS03485 indicated that 85.9% of the protein conformation was within the favored region. (C) ProSA analysis of the 3D-modeled structure of NGFG RS03485 indicated no significant deviation from typical native structures. The results generated display the Z-scores, which indicate the overall model quality, and energy plots, which indicate the local model quality. ProSA-web Z-scores of all protein chains in PDB are determined by X-ray crystallography (light blue) and nuclear magnetic resonance spectroscopy (dark blue) with respect to their length. The Z-scores of protein models were present in the range represented by the large black dot.

Homology modeling was performed for the shortlisted proteins were modeled to obtain a 3D structure. The homology modeling of the 3D structure of the proteins was performed using SWISS-MODEL (Fig. 2). Ramachandran plots, ERRAT, and ProSA were used to assess further the three sets of genes consistently utilized across many conditions of protein structure. Ramachandran plots provide an assessment of favorable regions. These plots thoroughly explore potential ψ and Ψ and combinations of steric conflicts between atoms using computer models of various dipeptides [92]. Based on the Ramachandran plot analysis, the protein's main chain conformation was more than 85% within the favored regions. The Z-score was used to assess the models' sequence-structure compatibility via ProSA [55]. Based on the Z-score predicted in ProSA, all the proteins’ 3D models were positioned inside the structural space of proteins as determined by X-ray crystallography. The ProSA analysis of the 3D-modeled structure of NGFG RS03485 indicated no significant deviation from typical native structures.

In the current study, computational biology was used to uncover novel therapeutic target candidates in the core proteome of 12 strains of *N. gonorrhoeae* by analyzing their protein sequences. Out of 12,300 core proteomes, one essential core protein with unique metabolic pathogen pathways was identified as a possible therapeutic target. Using computational databases and a subtractive genomics technique, we revealed the hitherto untapped potential of current computational databases and identified essential genes that may be evaluated as candidates for antibacterial drug discovery. Presumably, the prospective pharmacological targets identified from the *N. gonorrhoeae* core proteome will expedite the discovery of innovative anti-gonococcal medicines. The functional annotation of the proteins identified in this study provides a molecular understanding that will benefit the drug development process and potentially unravel a novel antibacterial target.

## Figures and Tables

**Table 1.** Summary of the analysis of identified proteins

Analysis	Identified proteins
Core proteome of 12 strains of *Neisseria gonorrhoeae*	12,300
Removal of non-paralogous proteins	944
Removal of proteins with <100 amino acids	476
Essentiality analysis	421
Virulent protein identification	120
Non-homology against the human proteome	101
Non-homology against gut microbiota	42
Broad spectrum analysis	42
Anti-target analysis	41
Host-pathogen interactions	27
KEGG metabolic pathway analysis	3
Subcellular localization	3
Druggability analysis	1

KEGG, Kyoto Encyclopedia of Genes and Genomes.

**Table 2.** Proteins involved only in unique pathogen-specific pathways

SNO	KO assignment	Protein ID	Pathway	Enzyme name
1	K00523	NGFG_RS03485	O-Antigen nucleotide sugar biosynthesis	CDP-4-dehydro-6-deoxyglucose reductase, E3 [EC:1.17.1.1]
2	K02535	NGFG_RS11485	Lipopolysaccharide biosynthesis	UDP-3O-[3-hydroxymyristoyl] N-acetylglucosamine deacetylase [EC:3.5.1.108]
3	K08324	NGFG_RS03515	Nicotinate and nicotinamide metabolism	Succinate-semialdehyde dehydrogenase [EC:1.2.1.16 1.2.1.24]

**Table 3.** Assessment of the drug target properties of the identified proteins

Protein ID	MW < 100 kDa	Mean pI <7.2	Hydrophobicity (GRAVY)–0.150 to –0.350	Length 400-600 (amino acids)	Signal peptide present likelihood > 0.5	TMMH > 1	O-glycosylation ≤ 1	N-glycosylation > 2	Aliphatic Index
NGFG_RS03485	36.6	6.21	–0.288	336	0.1126	0	1	3	82.56
NGFG_RS11485	33.9	5.21	–0.086	307	0.1358	0	0	2	98.18
NGFG_RS03515	49.3	6.3	–0.12	447	0.0049	0	2	3	84.14

MW, molecular weight; GRAVY, grand average hydrophobicity; TMMH, transmembrane helix.

## Supplementary Materials

Supplementary Table 1. Non-homologous human essential pathogens in unique metabolic pathways

Supplementary Table 2. Druggability analysis against the DrugBank database

Supplementary Table 3. Functional annotation of the identified protein
