# Supplementary material for: Antibiotic resistance in Neisseria gonorrhoeae: broad-spectrum drug target identification using subtractive genomics
Source: Genomics Inform. 2023 Mar 31;21(1):e5. doi: 10.5808/gi.22066 (PMC10085745; doi:10.5808/gi.22066)
Supplement: Supplementary Table 1. — Non-homologous human essential pathogens in unique metabolic pathways [file gi-22066-Supplementary-Table-1.pdf]

Supplementary Table 1. Non-homologous human essential pathogens in unique metabolic pathways

Human pathways	*Neisseria* pathways	Unique to *Neisseria* pathways
1. Glycolysis/gluconeogenesis	1. Glycolysis/gluconeogenesis	1. Butanoate metabolism
2. Citrate cycle (TCA cycle)	2. Citrate cycle (TCA cycle)	2. Oxidative phosphorylation
3. Pentose phosphate pathway	3. Pentose phosphate pathway	3. Alanine aspartate and glutamate metabolism
4. Pentose and glucuronate inter conversions	4. Galactose metabolism	4. Lysine biosynthesis
5. Fructose and mannose metabolism	5. Starch and sucrose metabolism	5. Arginine biosynthesis
6. Starch and sucrose metabolism	6. Amino sugar and nucleotide sugar metabolism	6. Histidine metabolism
7. Amino sugar and nucleotide sugar metabolism	7. Pyruvate metabolism	7. Tyrosine metabolism
8. Pyruvate metabolism	8. Glyoxylate and dicarboxylate metabolism	8. Phenylalanine tyrosine and tryptophan biosynthesis
9. Inositol phosphate metabolism	9. Propanoate metabolism	9. Lipopolysaccharide biosynthesis
10. Purine metabolism	10. Butanoate metabolism	10. O-Antigen nucleotide sugar biosynthesis
11. Glycine serine and threonine metabolism	11. Inositol phosphate metabolism	11. Nicotinate and nicotinamide metabolism
12. Valine leucine and isoleucine degradation	12. Oxidative phosphorylation	12. Folate biosynthesis
13. Lysine degradation	13. Purine metabolism	13. Tropane, piperidine and pyridine alkaloid biosynthesis
14. Arginine and proline metabolism	14. Alanine aspartate and glutamate metabolism	14. Novobiocin biosynthesis
15. Tryptophan metabolism	15. Glycine serine and threonine metabolism	
16. Glutathione metabolism	16. Valine leucine and isoleucine degradation	
17. Vitamin B6 metabolism	17. Lysine biosynthesis	
18. Retinol metabolism	18. Lysine degradation	
19. Porphyrin and chlorophyll metabolism	19. Arginine biosynthesis	
20. Streptomycin biosynthesis	20. Arginine and proline metabolism	
21. Aminoacyl t-RNA biosynthesis	21. Histidine metabolism	
22. The sulfur relay system base	22. Tyrosine metabolism	
23. Excision repair ABC	23. Phenylalanine metabolism	
24. Transporters	24. Tryptophan metabolism	
25. Phosphatidylinositol signaling system	25. Phenylalanine tyrosine and tryptophan biosynthesis	
26. Lysosome	26. Lipopolysaccharide biosynthesis	
27. Protein digestion and absorption	27. O-Antigen nucleotide sugar biosynthesis	
	28. Vitamin B6 metabolism	
	29. Folate biosynthesis	
	30. Porphyrin and chlorophyll metabolism	
	31. Tropane piperidine and pyridine alkaloid biosynthesis	
	32. Streptomycin biosynthesis	
	33. Novobiocin biosynthesis	
	34. Aminoacyl – t-RNA biosynthesis	
	35. Base excision repair	

36.	Phosphatidylinositol signaling system
