# Supplementary material for: Antibiotic resistance in Neisseria gonorrhoeae: broad-spectrum drug target identification using subtractive genomics
Source: Genomics Inform. 2023 Mar 31;21(1):e5. doi: 10.5808/gi.22066 (PMC10085745; doi:10.5808/gi.22066)
Supplement: Supplementary Table 2. — Druggability analysis against the DrugBank database [file gi-22066-Supplementary-Table-2.pdf]

Supplementary Table 2. Druggability analysis against the DrugBank database

Protein ID	Protein name	DrugBank ID	Drug name	Category	E-value	Bit score	Query length
NGFG RS03485	Benzoate 1,2-dioxygenase electron transfer component	DB03147	Flavin adenine dinucleotide	Approved	1.24E-31	120.168	336
	Bifunctional cytochrome P450/NADPH--P450 reductase	DB03440	N-hexadecanoylglycine	Experimental	4.19E-06	46.9802	336
		DB04257	Palmitoleic acid	Experimental			
		DB08086	N-[12-(1H-imidazol-1-yl)dodecanoyl]-L-leucine	Experimental			
		DB07861	(2R)-N-hydroxy-3-naphthalen-2-yl-2-[(naphthalen-2-ylsulfonyl)amino]propanamide	Experimental	6.62356e-102	299.671	
		DB01991	TU-514	Experimental			
NGFG RS11485	UDP-3-O-[3-hydroxymyristoyl] N-acetylglucosamine deacetylase	DB04257	Palmitoleic acid	Experimental			
		DB07355	3-(heptyloxy)benzoic acid	Experimental	1.41116e-42	146.747	307
		DB07536	N-{(1S,2R)-2-hydroxy-1-[(hydroxyamino)carbonyl]propyl}-4-{[4-(morpholin-4-ylmethyl)phenyl]ethynyl}benzamide	Experimental			
		DB08231	Myristic acid	Experimental			
		DB00157	NADH	Approved, nutraceutical			
	Retinal dehydrogenase 2	DB00162	Vitamin A	Approved, nutraceutical, vet-approved	2.60186e-65	217.238	
		DB00755	Tretinoin	Approved, investigational, nutraceutical			
		DB00157	NADH	Approved, nutraceutical			
NGFG_RS03515	Retinal dehydrogenase 1	DB00162	Vitamin A	Approved, nutraceutical, vet-approved			
		DB00755	Tretinoin	Approved, investigational, nutraceutical	2.59115e-63	211.46	

	DB04447	1,4-Dithiothreitol	Experimental		
	DB09462	Glycerin	Approved, investigational		
Gamma-aminobutyraldehyde dehydrogenase	DB04401	Betaine aldehyde	Experimental	5.17e-62	207.223
	DB00822	Disulfiram	Approved		
	DB00157	NADH	Approved, nutraceutical		
Aldehyde dehydrogenase, mitochondrial	DB02115	Daidzin	Experimental		
	DB00536	Guanidine	Approved		
	DB04381	Crotonaldehyde	Experimental		
	DB00727	Nitroglycerin	Approved, investigational	1.17932e-61	207.608
	DB00435	Nitric Oxide	Approved		
	DB09117	Paraldehyde	Approved, investigational		
NADP-dependent glyceraldehyde-3-phosphate dehydrogenase	DB02263	D-glyceraldehyde 3-phosphate	Experimental	5.56409e-61	204.527
	DB03461	Nicotinamide adenine dinucleotide phosphate	Experimental		
Succinate-semialdehyde dehydrogenase, mitochondrial	DB00534	Chlormerodrin	Approved, withdrawn		
	DB00157	NADH	Approved, nutraceutical	1.07817e-60	205.297
	DB00139	Succinic acid	Approved, nutraceutical		
	DB00313	Valproic acid	Approved, investigational		
	DB09072	Sodium oxybate	Approved		
