# Supplementary material for: Antibiotic resistance in Neisseria gonorrhoeae: broad-spectrum drug target identification using subtractive genomics
Source: Genomics Inform. 2023 Mar 31;21(1):e5. doi: 10.5808/gi.22066 (PMC10085745; doi:10.5808/gi.22066)
Supplement: Supplementary Table 3. — Functional annotation of the identified protein [file gi-22066-Supplementary-Table-3.pdf]

Supplementary Table 3. Functional annotation of the identified protein

Accession ID	Uniprot ID	Protein Name	GO	PFAM	PROSITE
NGFG RS03485	A0A6G7AIN5 NEIGO	2Fe-2S iron-sulfur cluster binding domain-containing protein	0009055; electron transfer activity<br>0046872; metal ion binding<br>0051537;2 iron, 2 sulfur cluster binding	PF00111<br>2Fe-2S iron-sulfur cluster binding domain<br>PF00175 Oxidoreductase NAD-binding domain<br>PF00970 Oxidoreductase FAD-binding domain	PS00197<br>2Fe-2S ferredoxin-type iron-sulfur binding region signature.<br>PS51085<br>2Fe-2S ferredoxin-type iron-sulfur binding domain profile.<br>PS51384 Ferredoxin reductase-type FAD-binding domain profile
